# Safe driving behaviors among taxi drivers: a predictive cross-sectional study based on the health belief model

**DOI:** 10.1186/s13690-020-00469-0

**Published:** 2020-09-16

**Authors:** Sakineh Dadipoor, Vahid Ranaei, Mohtasham Ghaffari, Sakineh Rakhshanderou, Ali Safari-Moradabadi

**Affiliations:** 1 Student Research Committee, Faculty of Health, Hormozgan University of Medical Sciences, Bandar Abbas, Iran; 2 Social Determinants in Health Promotion Research Center, Hormozgan Health Institute, Hormozgan University of Medical Sciences, Bandar Abbas, Iran; 3 School of Public Health and safety, Shahid Beheshti University of Medical Sciences, Tehran, Iran; 4 School of Public Health and safety, Shahid Beheshti University of Medical Sciences, Tehran, Iran; 5 Student Research Committee, School of Public Health and safety, Shahid Beheshti University of Medical Sciences, Tehran, Iran

**Keywords:** Taxi drivers, Safety, Behavior

## Abstract

**Background:**

The purpose of this study was to predict safe driving behaviors among taxi drivers of Tehran based on the constructs of health belief model.

**Methods:**

This descriptive-analytical study was performed on 450 taxi drivers in Tehran using multi-stage sampling. Data were analyzed through SPSS software version 18 using Pearson correlation coefficient and multivariate regression analysis. The *P* < 0.05 was considered statistically significant.

**Results:**

Among the constructs of health belief model, perceived severity, perceived benefits and self-efficacy, had significant and direct relationship and perceived barriers had a significant and reverse relationship with safe driving behaviors. According to the results, the constructs of health belief model predicted 17.3% of safe driving behaviors. Self-efficacy was the strongest determinant of safety behaviors (0.362 CI 0.098–0.625).

**Conclusions:**

Increasing self-efficacy, reducing perceived barriers and highlighting benefits for the purpose of accepting safe behaviors can be considered as a principle in driving education and training. Also, increasing the perceived severity of adverse outcomes of RTAs and the susceptibility to these outcomes will lead to higher levels of safe driving behaviors.

## Background

Every year, nearly 1.2 million people die and more than 50 million people get injured as a result of road traffic accidents (RTA). Ninety percent of these figures belong to the low or middle income countries [1]. Several studies have shown that human factors, which are known as hazardous or unsafe behaviors, play a vital role in road traffic accidents [2–4]. A wide range of these behaviors has been reported in various studies that includes driving behaviors (such as violation of driving rules and speed limit) and disturbance in driving skills (such as fatigue, lack of concentration and physical disabilities, etc.) [5]. Among other unsafe behaviors that can lead to RTAs are drink driving, non-use of seatbelts while driving, and mobile phone calls while driving [6, 7]. Knowledge, belief, and attitude toward high risk driving behaviors and driving experience are also important aspects of hazardous behaviors [8–10]. In Iran, due to the state of public transportation, taxis play a key role in the urban transportation market and provide appropriate services for passengers. According to the statistics of Traffic and Transportation Institute of Tehran municipality, the share of public transportation in urban trips in the city of Tehran is about 48.9% from which, 7.2% belong to rail transport (Metro), 15.5% belong to buses, 24.3% belong to taxis and 2% belong to minibuses [11]. More experienced drivers, who perceive themselves professional drivers, tend more to show risky behaviors in order to serve one passenger as fast as possible and serve the next, and, thus, earn more money [12]. The mortality rate among taxi drivers is about 14.9% versus 3.3% in other occupations. Also, violent acts among taxi drivers who may not have a passenger for a day is 3.7 per 10,000 compared to 2.4 per 10,000 in other occupations [13].

According to statistics of WHO, a large number of people use vehicle on daily basis, all of whom are at risk of road traffic accidents. Most of these accidents are caused by drivers’ unsafe behavior. Although RTA may be a common event, it is both predictable and preventable [14–17]. Therefore, it is very important to select a model that can predict the psychological variables associated with unsafe behaviors among taxi drivers. One of the suggested models in this regard is the health belief model, which consists of several constructs including perceived susceptibility (person feels that doing unsafe behaviors increases the probability of accident), perceived severity (person feels that accident will lead to serious injuries to him and his life), perceived benefits (person understands the advantages of safe behaviors), perceived barriers (person understands the barriers to safe behaviors), and perceived self-efficacy (ensuring the ability to perform safe driving behaviors) [18]. Aghamollah et al. conducted a study to predict safe driving behaviors in Bandar Abbas using health belief model [19]. Some studies have also implemented educational interventions based on health belief model in order to reduce unsafe driving behavior of taxi drivers [18, 20].

Driving behavior is part of cultural behaviors of people in society because it is related to values, habits, attitudes and other factors. Therefore, we can reduce the number of accidents only when risky driving behavior and its effective factors in a particular culture have been reviewed and studied. Thus, considering the abovementioned issues and the fact that few studies have been carried out on taxi drivers in Iran, and also taking into account the dangers of unsafe behaviors for taxi drivers, this study was conducted to predict unsafe behaviors among taxi drivers of Tehran using health belief model.

## Method

### Study design

This cross-sectional study was conducted on taxi drivers of Tehran. Information on the total number of taxis working in Tehran streets, the type of cars involved and all service routes within the city was obtained from Tehran head office of taxi drivers. Due to the better discipline of line taxi drivers than others, particularly in working hours per day, from among 77,955 taxis in Tehran, 53,947 line taxis active in 591 distinct routes were selected as the sample. Since the community of Tehran taxi drivers is limited, Cochran’s formula for limited society was used for the sample size, and then using Cochran correction formula for limited population with the assumption of 0.5 ratio of high risk behavior, the sample size was decided to be 450. Assuming that 9 taxies should be studied in every route line, a total number of 50 routes needed to be selected. Selection of taxi route lines for the study was done using random tables from the existing 591 route lines in Tehran. Then, 9 taxis for each route line were selected randomly at different hours of the day. It should be noted that, of the 9 taxis selected, 5 departing taxies and 4 arriving taxis were examined.

### Entry and exit criteria

Reading and writing skills, having 1 year of driving experience as a taxi driver, and living in Tehran were among the entry criteria, and also partial completion of the questionnaire was the criterion for leaving the study.

### Instrument

The data collection instrument in this study was a researcher-made self-rating questionnaire made up of two parts:

#### Part one

This section contained demographic information such as age, education, driving history, history of working as a taxi driver, history of fines due to driving violations and history of road traffic accidents.

#### Part two

This section was related to the constructs of health belief model and safe driving behaviors (Table 1).

**Table 1:** Description of study instrument

Construct	Number of items (Format)	Scoring (range)	Validity (Cronbach’s alpha)	Example of items
1)Perceived susceptibility: It refers to subjective assessment of risk of developing a health problem	5 items/ 5 point Likert Scale	Strongly Disagree = 1, Disagree = 2, No idea = 3, Agree = 4, Strongly Agree = 5 (5–25)	0.79	If I drive with unauthorized speed, I’m likely to crash
2) Perceived severity: It refers to the subjective assessment of the severity of a health problem and its potential consequences.	5 items/5 point Likert Scale (strongly disagree- strongly agree)	SD = 1, D = 2, NI = 3, A = 4, SA = 5^a^ (5–25)	0.84	If I have driving accident, I will suffer from long-term complications.
3) Perceived benefits: Health-related behaviors are also influenced by the perceived benefits of taking action.	6 items/5 point Likert Scale (strongly disagree- strongly agree)	SD = 1, D = 2, NI = 3, A = 4, SA = 5 (6–30)	0.81	Avoiding hazardous driving behaviors will prevent personal and financial losses.
4) Perceived barriers: It refers to barrier to taking health-related actions.	5 items/5 point Likert Scale (strongly disagree- strongly agree)	SD = 1, D = 2, NI = 3, A = 4, SA = 5 (5–25)	0.85	The temptation to transfer more passenger makes me stop driving safely.
5) Self-efficacy: It refers to an individual’s perception of his or her competence to successfully perform a behavior	4 items/5 point Likert Scale (strongly disagree- strongly agree)	SD = 1, D = 2, NI = 3, A = 4, SA = 5 (4–20)	0.76	The insistence of passengers does not make me to undertake risky driving behaviors.
6) Safe behavior: It refers to health-related behaviors and practices.	32 items / 4 point	never = 1, sometimes = 2, most of the time = 3, Always = 4) (32–128)	–	- Use mobile phone while driving - Exchanging money with passengers while driving

^a^*SD* Strongly Disagree, *D* Disagree, *NI* No Idea, *A* Agree, *SA* Strongly Agree

To determine the reliability of the questionnaire (clarity, simplicity and relevance of the questions with the objectives of the study), it was given to 20 taxi drivers with conditions similar to the entry criteria. A numerical score of less than 0.79 indicated the removal of item from the tool. In this study, no item was removed from the original questionnaire, and only a few were corrected. The questionnaire was also given to 5 experts in the fields of health education, safe behaviors and traffic and their comments were used to amend the questionnaire.

### Data analysis

After collecting the data, they were entered into SPSS-16 software and were described using mean and standard deviation. Also, to determine the correlation between the constructs of health belief model and safe driving behaviors, Pearson’s correlation coefficient was used and to predict safe driving behaviors within the frameworks of health belief model constructs, multivariate regression analysis was used. The *P* < 0.05 was considered statistically significant.

## Results

In total, 450 individuals participated in this study. One person was excluded from the study due to partial completion of the questionnaire. The mean age of the subjects was 44.07 ± 11.15 years, their mean driving experience was 17.26 ± 10.74 years and their mean driving experience with taxi was 9.68 ± 7.29 years. From the participants, 16 (3.5%) were illiterate, 398 (88.5%) had basic education and 36 (8%) had academic education. Also, 142 (31.5%) drivers had a history of fines and 106 (23.5%) had a history of accident while transferring passenger.

The frequency distribution of health belief model constructs and the adoption of preventive behaviors are shown in Table 2. Results of this table show that, the subjects obtained a lower score for the structures of perceived barriers and safe driving behaviors compared to other structures. Also, the constructs of perceived susceptibility and perceived benefits accounted for the highest score among all variables in this study.

**Table 2:** Mean score and standard deviation of health belief model constructs and safe driving behaviors (*N* = 440)

Variable	Mean ± SD	Range of obtained scores	The percentage of score obtained from the maximal score
Perceived Susceptibility	18.72 ± 2.39	5–25	93.63
Perceived Severity	16.35 ± 2.88	5–25	81.78
Perceived Benefits	22.29 ± 2.83	6–30	92.88
Perceived Barriers	12.01 ± 5.77	5–25	60.07
Self-Efficacy	13.32 ± 2.24	4–20	83.30
Safe Driving Behavior	54.27 ± 5.46	32–128	56.54

The findings showed a direct relationship between all constructs of health belief model, except for the structure of perceived barriers, and adopting preventive behaviors. On the other hand, there was no significant relationship between the construct of perceived susceptibility and safe driving behaviors (Table 3**)**.

**Table 3:** The matrix of Pearson’s correlation coefficient of the health belief model constructs and safe driving behaviors

Variable	Perceived Susceptibility	Perceived Severity	Perceived Benefits	Perceived Barriers	Self-Efficacy	Driving Behavior
Perceived Susceptibility	1					
Perceived Severity	.143^a^ 0.002	1				
Perceived Benefits	.273^a^ .000	.416^a^ .000	1			
Perceived Barriers	−.176^a^ .000	.279^a^ .000	−.173^a^ .000	1		
Self-Efficacy	.166^a^ 000	.290^a^ .000	.321^a^ .000	.073 .120	1	
Driving Behavior	.039 .478	.163^a^ .002	.300^a^ .000	−.223^a^ .000	.247^a^ .000	1

^a^Correlation is significant at 0.01 level

Table 4 shows the results of multivariate regression analysis to determine the predictive constructs of safe driving behaviors, and also the predictive value of behavior by these structures in the health belief model. According to the results of this table, constructs of health belief model predicted 17.3% of safe driving behaviors. Among the studied constructs, perceived severity, perceived benefits, perceived barriers and self-efficacy were highly predicting the safe driving behavior. Self-efficacy was the strongest determinant of the safety behavior.

**Table 4:** Multivariate regression analysis of predictive structures for adopting safe driving behaviors based on health belief model

Variable	Unstandardized Coefficients		t	Sig.*	95.0% Confidence Interval		R square
	B	Std. Error			Lower Bound	Upper Bound	
**(Constant)**	43.954	2.825	15.561	.000	38.398	49.511	0.173
**Perceived Susceptibility**	−.152	.110	−1.380	.169	−.369	.065	
**Perceived Severity**	.303	.119	2.550	.011	.069	.536	
**Perceived Benefits**	.298	.111	2.691	.007	.080	.516	
**Perceived Barriers**	−.281	.057	−4.953	.000	−.392	−.169	
**Self-Efficacy**	.362	.134	2.697	.007	.098	.625	

*Independent variables entered the regression model by the simultaneous method

## Discussion

The purpose of this study was to predict the safe driving behaviors of taxi drivers in Tehran based on the health belief model. The findings showed a strong correlation between the constructs of model (except for perceived susceptibility) and safe driving behaviors. Self-efficacy was the strongest determinant of safety behavior.

As the results showed, self-efficacy was the strongest predictor of safe behavior among taxi drivers. Studies in this regard confirmed the relationship between self-efficacy and safe driving behaviors [20, 21]. Razmara et al. (2018) reported that drivers with lower emotional self-efficacy had higher hazardous driving behaviors than drivers with higher emotional self-efficacy. Contrary to the findings of present study, a study showed no significant relationship between self-efficacy and safe behaviors of taxi drivers [19]. To some extent, these contradictions can be attributed to the differences in the personality and demographic characteristics of the target community and the dominant culture of different countries. Individuals with high self-efficacy seem to feel more confident and competent than those with low self-efficacy, and therefore are less likely to behave negatively. It can also be argued that when people believe they can drive safely against road accidents and this behavior can reduce their health risks, they are less likely to drive dangerously. The construct of self-efficacy should be considered more closely because it can be strongly linked to safe driving behaviors, as knowing the reasons for doing a behavior alone is not enough, but the person must fell capable of doing the behave.

In this study, perceived susceptibility was not a predictor of safe behaviors among taxi drivers. In line with this study, a study showed that perceived susceptibility is not a predictor of safety behavior among taxi drivers [19]. Unlike the present study, studies have acknowledged the role of perceived susceptibility in promoting safe behaviors [22, 23]. The reason for this contradiction may be attributed to the study population of present study (taxi drivers), probably because taxi drivers consider themselves to be trained and professional drivers, therefore they are not susceptible to accident. The effective and successful prevention of accidents depends on the actual information about the susceptibility to and the likelihood of the risk, so promoting awareness often results in perceived susceptibility and ultimately leads to safe behaviors.

In the present study, the perceived severity was found to be a predictor of safe behavior among taxi drivers. In line with this finding, a perceived threat from high-risk driving behaviors was associated with a reduction in these behaviors in another study [23]. Other studies refer to threat evaluation by drivers as a reason for the reduction of hazardous behaviors such as carelessness driving [24, 25]. Also, Şimşekoğlu et al. showed that perceived threat leads to cautious behaviors, including seat belts use and speed reduction. The perceived threat in present study seemed to have encouraged the drivers to increase their safe driving behaviors. Contrary to the findings of present study, perceived severity in some studies did not predict safe behavior among drivers [19, 26, 27]. The reason for this contradiction can be attributed to the personality traits, cultural differences, and differences in purpose, instrumentation, and type of the studied subjects.

The current study showed that perceived benefit was a predictor of safe driving behavior. Consistent with this finding, in another study perceived benefit was found to be a predictor of safe behavior among taxi drivers [19]. But in the Gras et al. study (2007), perceived benefit was not a predictor of using seatbelts [18]. This difference can be attributed in part to the differences in the study design and objectives and the personal and cultural characteristics of the subjects. The average score of perceived benefits among taxi drivers in the current study was due to the fact that, the drivers had a high perceptions of benefits of safe behaviors. It means that, one comes to believe that using safety belt can reduce the severity of injuries in an accident, and this action is beneficial for him/her. Therefore, it seems that highlighting benefits through training and education is necessary for drivers to promote safety behaviors.

In this study, there was a significant and reverse relationship between the perceived barriers and safe driving behaviors. In line with the findings of present study, a study showed that perceived barriers after guideline is the most powerful predictor of safe behavior among taxi drivers [19]. Many studies are in line with these findings [28–30]. It seems that in order to increase safety behaviors, obstacles should be removed or reduced. For example, the quality of roads is one of the obstacles to safe behavior among taxi drivers. In a study, taxi drivers braked suddenly or quickly turned to avoid falling into the holes on the roads, which could have caused problems for the following car [1]. Other barriers include the lack of facilities and old or worn out vehicles, because driving with these cars can be difficult due to their low level of safety. Therefore, accurate identification of perceived barriers and removal of them to promote safe driving behaviors is essential.

### Limitations

One of the limitations of present study was the self-reporting of the questionnaires, which might have affected the accuracy of the results. Due to the limitations of human studies and not being able to directly observe the drivers’ behaviors, the results must be generalized with caution. Another limitation of this study could be its inability to be generalized to other drivers, because the participants in this study were taxi drivers, thus the results should not be generalized to the drivers of other vehicles. The cross-sectional nature of this study was another limitation. It is suggested that in future studies, educational interventions should be conducted with long-term follow-up, and also direct observation method should be used to measure performance.

## Conclusion

The results of this study confirmed the effectiveness of health belief model in predicting unsafe driving behavior. The use of this model should be closely considered as a framework for developing educational programs to improve driving behaviors and reduce road traffic accidents. Increasing self-efficacy, reducing perceived barriers and highlighting benefits in order to accept safe behavior can be considered as a principle in driving training and education. Also, raising awareness about the perceived severity of the adverse outcomes of RTAs and susceptibility towards these outcomes can lead to the adoption of higher levels of safe driving behaviors.

## Data Availability

The datasets used and analyzed during the current study are available from the corresponding author on reasonable request.

## Abbreviations

RTA: Road traffic accidents
WHO: World Health Organization
